# “Burnt by the scorching sun”: climate-induced livelihood transformations, reproductive health, and fertility trajectories in drought-affected communities of Zambia

**DOI:** 10.1186/s12889-021-11560-8

**Published:** 2021-08-03

**Authors:** Joseph G. Rosen, Drosin Mulenga, Lyson Phiri, Natasha Okpara, Caila Brander, Nachela Chelwa, Michael T. Mbizvo

**Affiliations:** Population Council, Lusaka, Zambia

**Keywords:** Drought, Sexual and reproductive health, Fertility, Climate change, Qualitative research, Sub-Saharan Africa

## Abstract

**Background:**

Climate-induced disruptions like drought can destabilize household and community livelihoods, particularly in low- and middle-income countries. This qualitative study explores the impact of severe and prolonged droughts on gendered livelihood transitions, women’s social and financial wellbeing, and sexual and reproductive health (SRH) outcomes in two Zambian provinces.

**Methods:**

In September 2020, in-depth interviews (*n* = 20) and focus group discussions (*n* = 16) with 165 adult women and men in five drought-affected districts, as well as key informant interviews (*n* = 16) with civic leaders and healthcare providers, were conducted. A team-based thematic analysis approach, guided by the Framework Method, was used to code transcript text segments, facilitating identification and interpretation of salient thematic patterns.

**Results:**

Across districts, participants emphasized the toll drought had taken on their livelihoods and communities, leaving farming households with reduced income and food, with many turning to alternative income sources. Female-headed households were perceived as particularly vulnerable to drought, as women’s breadwinning and caregiving responsibilities increased, especially in households where women’s partners out-migrated in search of employment prospects. As household incomes declined, women and girls’ vulnerabilities increased: young children increasingly entered the workforce, and young girls were married when families could not afford school fees and struggled to support them financially. With less income due to drought, many participants could not afford travel to health facilities or would resort to purchasing health commodities, including family planning, from private retail pharmacies when unavailable from government facilities. Most participants described changes in fertility intentions motivated by drought: women, in particular, expressed desires for smaller families, fearing drought would constrain their capacity to support larger families. While participants cited some ongoing activities in their communities to support climate change adaptation, most acknowledged current interventions were insufficient.

**Conclusions:**

Drought highlighted persistent and unaddressed vulnerabilities in women, increasing demand for health services while shrinking household resources to access those services. Policy solutions are proposed to mitigate drought-induced challenges meaningfully and sustainably, and foster climate resilience.

## Background

Climate change is among the greatest threats to public health in modern times [1, 2]. The World Health Organization (WHO) estimates that between 2030 and 2050, climate change will be responsible for 250,000 excess annual deaths (from malnutrition, malaria, diarrhea, and heat stress) and US$ 2–4 billion in direct health costs per year by 2030 [3]. Environmental disruptions attributed to climate change are well-documented, ranging from droughts to flash floods to extreme or prolonged heat [4, 5]. Climate-induced disruptions can be destabilizing forces for households and communities, resulting in food insecurity, unemployment, environmental degradation, and health threats [6–8]. In low and middle-income countries (LMICs), those most affected by extreme and recurrent environmental disruptions are often poor and rural [9]; these climate-affected populations also tend to be marked by poorer sexual and reproductive health (SRH) outcomes [10–12]. Due to underlying inequities restricting their access to social and material assets, women and girls are disproportionately impacted by ecological and economic stressors attributed to climate change. Uninterrupted access to SRH services, including family planning, remain key considerations, despite climate change-induced disruptions.

Given women and girls’ heightened vulnerability to climate change, particularly in LMIC settings, these environmental disruptions are likely to affect women’s SRH, from fertility intentions to birth outcomes. Reduced school attendance for girls [13], early marriage [14], and male out-migration [15] have been documented in climate-affected populations. Nonetheless, the impact of climate change on SRH outcomes has received insufficient attention in the literature. Some evidence has emerged highlighting the deleterious impact of climate change on women’s health; for example, studies in South Asia have attributed increased risk of preterm birth and preeclampsia to environmental changes, specifically increases in ambient temperature and water salinity, respectively [16–18]. Climate volatility can also destabilize health infrastructure by rendering essential services inaccessible due to inclement weather, increasing the risk of maternal complications [19]. Sexual and gender-based violence is frequently reported during forced migration events, which have received increased attention in the context of climate change [20, 21]. Major gaps persist with regards to understanding the mechanisms through which climate change may exact a toll on SRH outcomes, both directly and indirectly.

In Zambia, climate change is responsible for numerous environmental hazards, including more frequent and intense seasonal droughts, increased valley temperatures, prolonged dry spells, and flash flooding [22]. Zambia’s agricultural sector supports roughly 85% of the country’s population, employing 52% of the country’s working-age population—a majority of whom are women and rural-dwelling residents [23]; rainfall anomalies and other climate variations threaten this mostly rain-fed industry. The Government of the Republic of Zambia (GRZ) has projected a $5 billion GDP deficit over a 10–20-year period due to the impact of climate change on agricultural productivity, poverty, energy production, healthcare costs, and loss of natural environments [24]. As much of Zambia’s electricity is hydroelectric, rainfall anomalies will impact electricity access, particularly in rural areas most impacted by drought. The projected energy crisis is also expected to exacerbate environmental degradation, as the increased demand for charcoal production will motivate continued deforestation [25].

Given the potential synergistic impacts of this growing climate crisis and gender-based inequities on the SRH outcomes of women and girls, there is an urgent need to interrogate the intersection of climate change, gender, and SRH in Zambia, more closely. The economic impacts of climate volatility on rural communities, whose livelihoods are inextricably linked to agriculture and farming, are well-documented in sub-Saharan Africa [26–28]. Drought, specifically, threatens agricultural productivity, resulting in heightened food insecurity and diminished household incomes; these processes can catalyze other downstream risks, like early marriage and transactional sex, associated with poverty. While disruptions to essential health services are anticipated with extreme weather events [29, 30], the impact of severe and persistent climate irregularities, like drought, on provision of routine SRH services (i.e., contraception, family planning) is unknown.

In response to these evidence gaps, this qualitative study explored the impact of severe and prolonged drought in two Zambian provinces on household livelihoods, women’s social and economic wellbeing, SRH service utilization, and fertility intentions. Through in-depth interviews (IDIs), focus group discussions (FGDs), and key informant interviews with community members, civic leaders, and healthcare providers in drought-affected communities, the study interrogated the gendered impacts of climate change and household climate adaptation strategies; shifting SRH needs among women in the context of climate change; adaptation strategies adopted within households and communities in response to drought; and recommendations for public and private sector interventions to foster climate resilience.

## Methods

### Setting

**Fig. 1:**
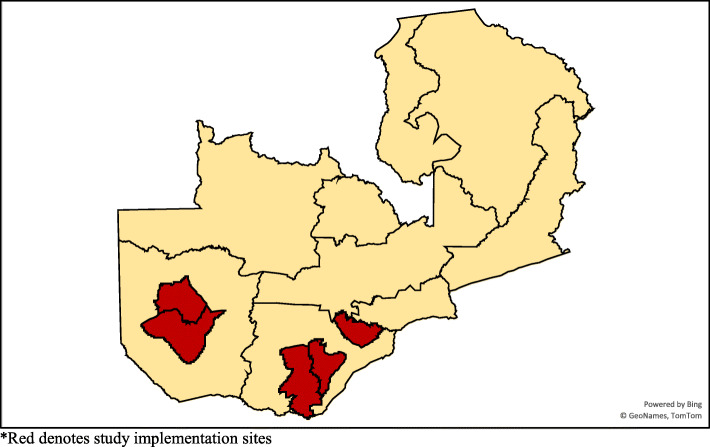
Study implementation districts in Zambia, September 2020.*. *Red denotes study implementation sites

The study was implemented in five districts in Zambia’s Southern and Western Provinces: Choma, Mazabuka, Mongu, Kalomo, and Senanga (see Fig. 1). Livelihoods in the districts are largely agricultural, and reduced rainfall has led to crop shortages in recent years. Western and Southern Provinces are located in semi-arid regions, with mean annual rainfall ranging between 600 mm–800 mm [31]. Western Province, Zambia’s largest administrative jurisdiction (with 14 districts), is where the country’s logging and rice industries are concentrated. Southern Province is a maize- and sugar-producing region of Zambia and home to the country’s premier tourist attraction, Mosi-oa-Tunya (Victoria Falls), which is shared with Zimbabwe. As throughout Zambia, a majority (~ 85%) of households employed in the agriculture sector in these districts are smallholder farms, with maize being the dominant agricultural crop, grown by over 82% of households [32, 33]. Both provinces have experienced rainfall anomalies over the last decade, including a particularly profound drought beginning in 2018–19 that has persisted through 2020–21. Limited infrastructure and support for climate-responsive agricultural practices have also rendered these districts particularly susceptible to poorer crop yields in times of drought. Fewer than half (45%) and 40% of Zambian farmers do not use fertilizer on their fields and plant hybrid maize seeds, respectively, rendering agricultural outputs particularly vulnerable to rainfall anomalies [34].

### Recruitment

Study participants included adult men and women from drought-affected communities; Provincial-level Ministry of Health (MoH) personnel involved with SRH service provision; Ministry of Agriculture (MoA) staff engaged in climate mitigation and adaptation activities, including Disaster Management and Mitigation Unit (DMMU) personnel; representatives from non-governmental organizations (NGOs) supporting food relief and climate adaption efforts; local political and religious leadership (i.e., chiefs, church leaders); and facility-based healthcare providers. Agriculture camp extension officers based in the study communities, who train farmers on livestock management and crop production strategies, identified eligible adult men and women for study enrollment and facilitated introductions of study staff to prospective participants. Extension officers purposively identified and selected adult men and women whom they believed would contribute the most meaningful, rich information to the qualitative data collection activities. Eligible participants included individuals: aged 18 years and older; proficient in English and/or one of three Zambian languages (Tonga, Lozi, Nyanja); and providing written informed consent to participate. Recruitment of climate change stakeholders and community members helped elicit a heterogeneity of perspectives, supporting rich narrative integration and triangulation across participant subgroups and study sites.

### Data collection

In September 2020, the research team facilitated 16 sex-segregated FGDs, each 120–180 min in duration and with an average of eight participants, with 145 men and women. IDIs (~ 60 min in duration) were conducted with: 20 adult women aged 18–49 who resided in the study district for at least a year; 16 stakeholders (i.e., religious leaders, local chiefs, provincial-level representatives from NGOs and government ministries), including seven healthcare providers. Data collection instruments were semi-structured in nature, allowing interviewers and FGD moderators to inductively pursue and probe on insights emerging from participant narratives not explicitly covered in the field guides. IDIs and FGDs were facilitated by six research assistants experienced in qualitative methods, who participated in a week-long intensive training covering study implementation, field guide piloting, and research ethics. Data collectors were supervised by two study coordinators (DM, LP) and a research manager (NC)—all three of whom are native Zambian researchers with extensive experience conducting qualitative studies.

IDI and FGD guides broached a variety of topics, including: participants’ understanding of climate change, specifically causes of drought; perceived impact of drought on household livelihoods, gender dynamics, and SRH, including fertility intentions and contraceptive use; and awareness of existing, and suggestions for future, interventions supporting household and community adaptation to drought. Given that field guides covered potentially sensitive SRH topics (i.e., contraceptive use, fertility intentions), additional individual interviews were conducted with women as a complement to the FGDs. Compared to community member interviews, IDIs with stakeholders solicited perspectives on service provision in the context of climate change, including availability and implementation of climate mitigation and adaptation interventions—to which some community members may not be privy. All data collection activities were conducted in private settings, including school classrooms, offices in health facilities, churches, private vehicles, or outdoor spaces (e.g., underneath trees). Interviewees and focus group discussants received 60 Kwacha (~$3 USD) for their participation.

### Analysis and interpretation

IDIs and FGDs were conducted in English, Lozi, Nyanja, and/or Tonga, audio-recorded, transcribed verbatim, and translated into English, when necessary. After close reading of transcripts and field notes, four study staff (JGR, DM, LP, NO), with guidance from study managers (NC, MTM), developed a codebook capturing deductive (i.e., from topics probed in the semi-structured guides) and inductive (i.e., emerging from participant narratives) themes. Applying the Framework Method [35], study staff collapsed topics from the semi-structured field guides into discrete code families; themes emerging in IDIs and FGDs were identified and classified as sub-codes nested within code families. The draft codebook was pilot tested with a sample of transcripts to assess breadth of themes captured and consistency in code application.

After reconciling coding discrepancies and finalizing the codebook, study staff applied codes to text segments from IDI and FGD transcripts using the codebook programmed in NVivo 12 (QSR International©, Melbourne, Australia). All transcripts were coded by at least one analyst. After coding all transcripts, analysts first reviewed coding output using software-facilitated code queries and reports, identifying salient themes and patterns from coded text segments to support data synthesis and interpretation. Using constant comparison [36], coded text segments were then charted manually into an analytic matrix, which helped visualize relationships between key themes derived from the coding framework [37]. This approach also facilitated comparison of themes emerging in IDIs and FGDs across key strata, including participant subgroup (i.e., men, women, stakeholders) and community type. Memo-writing throughout the analysis process, from initial close reading to post-coding data charting, supported further distillation and crystallization of thematic patterns.

## Results

A demographic profile of participants in FGDs, IDIs, and key informant interviews (*N* = 181) is presented in Table 1. The median age was 34 years (range: 19–73 years), and most were women (56%). A majority were married (71%) and employed in the agricultural sector (60%).

**Table 1:** Demographic profile of study participants, by data collection method (*N* = 181)

	Focus Group Discussions (*N* = 145)	In-Depth Interviews (*N* = 20)	Key Informant Interviews (*N* = 16)
**Age, in years** (median, range)	34 (19–49)	32 (22–44)	31 (25–73)
**Gender**			
Women	75 (52%)	20 (100%)	7 (44%)
Men	70 (48%)	–	9 (56%)
**Province**			
Southern	73 (50%)	9 (45%)	10 (63%)
Western	72 (50%)	11 (55%)	6 (37%)
**Marital status**			
Married	106 (73%)	11 (55%)	11 (69%)
Unmarried	39 (27%)	9 (45%)	5 (31%)
**Education**			
Primary or less	75 (52%)	8 (40%)	–
Secondary or higher	70 (48%)	12 (60%)	16 (100%)
**Occupation**			
Agriculture	102 (70%)	7 (35%)	–
Business/sales	20 (14%)	4 (20%)	–
Education	1 (1%)	2 (10%)	–
Healthcare	–	1 (5%)	7 (44%)
Domestic labor	12 (8%)	2 (10%)	–
NGO/professional	–	–	9 (56%)
Other/unemployed	10 (7%)	4 (20%)	–
**No. of children** (median, range)	4 (0–13)	3 (0–13)	1 (0–15)

***“The more we tamper with the trees, the more things change”: Characteristics and causes of climate change.***

Participants unanimously described climate change as an erratic, unpredictable, and destabilizing force impacting all facets of their lives, from crop yields to their health. The most frequently reported manifestation of climate change was drought, which was characterized by extreme heat and infrequent rains during periods historically characterized by heavy rainfall.

*Last year, rain started in November. By December...the rains had gone, and we delayed in planting. In January, it rained again, but only in intervals, and that affected the growth of most crops.* — Woman, FGD, Mazabuka.

*You have fewer rain days...You have high temperatures...hitting around 25–26 degrees [centigrade]. Then, you have pests that thrive in warm temperatures and affect food production. The water lagoons where people used to do their fishing have dried up.* — Church Leader, IDI, Mongu.

For most participants, climate change was manmade and attributed to human interactions with the environment, specifically deforestation. Men, in particular, narrated a cyclical process whereby reduced economic prospects attributed to drought drove men to industries (i.e., timber logging, charcoal production) contributing to deforestation, which subsequently worsened climate change processes.

*They have cut down a lot of trees to make charcoal. When this is done, it causes drought. These same trees also supply us with oxygen for breathing...We tamper with our forest because we want to find food to feed our families, but the more we tamper with the trees, the more things change.* — Man, FGD, Senanga.

Few participants, primarily women in rural districts, discussed spiritual or celestial origins to changing rainfall patterns in their communities.

*Rain comes from God, and there is a song that talks about God being the owner of the rains. We do not know what God’s plans are. Maybe he did not want to give us rains, so that His children can change their ways.* — Woman, FGD, Kalomo.

***“If I plant, the crops will come out well but later will be burnt by the scorching sun”: Impact of drought on livelihoods and wellbeing.***

Participants explained that severe droughts resulted in poor agricultural output, as their crops failed to mature. Drought was particularly deleterious in these study communities, as most participants relied on small-scale agriculture to feed and support their families. Drought also rendered water inaccessible to many households, who depended on wells (now dried up) for their farms, household consumption, and animal-rearing. Some households were forced to sell their livestock when they could no longer care for their animals, and more livestock deaths were reported during periods of drought.

*We can plant and yet have a poor harvest. It is too hot, and there is no rain...There are no means to find money*...*My children used to eat. But now, if I plant, the crops will come out well but later will be burnt by the scorching sun*. — Woman, IDI, Senanga.

### Gendered divisions of labor and employment

Economic strife and food insecurity propelled men and women to pursue alternative sources of employment to generate household income lost to drought. Prior to drought, participants described structured, patterned divisions of labor by gender. Men primarily held jobs involving manual labor (e.g., farming, construction, fishing), while women divided their time between micro-entrepreneurial activities and household obligations, like cooking, cleaning, and child-rearing. Even in dual breadwinning households, where men and women participated equally in non-household labor, employment opportunities (and, therefore, potential earnings) were highly gendered, with men managing small-scale businesses (e.g., planting and tilling farms) and delegating women to sell commodities, like produce and fish, generated from micro-enterprises.

*There are some activities that only can be done by a man or woman, so we allocate based on who can manage to do a certain activity. Clearing the bushes in the cassava fields is done by men, while digging up cassava is done by women...What is supposed to be done by a man should be done by a man, and what is supposed to be done by a woman should be done by a woman.* — Man, FGD, Mongu.

*A man orders goats and takes them home, so the wife can take them to Lusaka [capital city] for sale. When she gets back, she gives the money to the husband, who goes to order goats again, while she remains taking care of the home.* — Woman, FGD, Mazabuka.

As drought virtually eliminated viable employment opportunities in small-scale agriculture, demand for alternative income sources outstretched the available supply. While employment opportunities were available in burgeoning industries like timber logging and charcoal burning for men and beer brewing for women, some of these opportunities were seen as temporary or seasonal, offering little in terms of employment security or stable income.

**Fig. 2:**
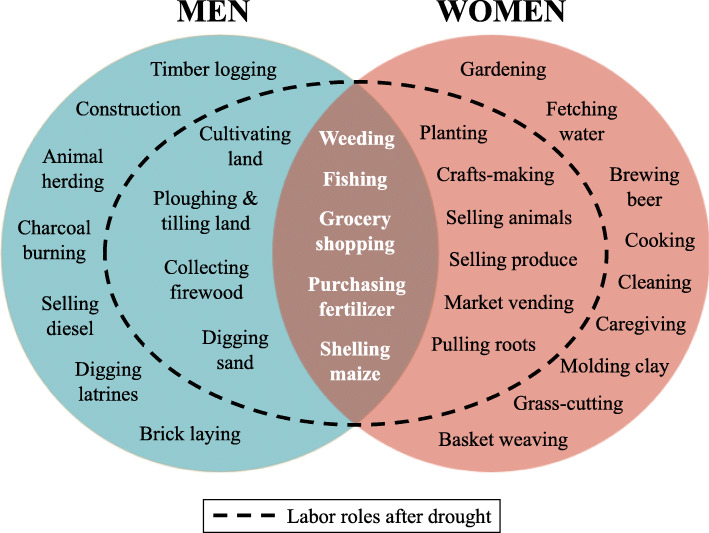
Gendered division of labor before and after drought

Participant narratives suggested that drought blurred a historically gendered division of employment roles. A shrinking labor market forced men and women to prioritize potential earnings over workforce preferences, pushing women into jobs with heightened manual labor demands and men into labor sectors traditionally dominated by women (see Fig. 2).

*I always go to the mountains in Gwangwazu...to collect wild tubers. I walk for long distances, and at times, I reach home late in the night. Then in the morning, I take the tubers for sale. I have never done this in my life, but I started doing that when I saw the challenges of drought, like how I was going to feed my children.* — Woman, FGD, Kalomo.

### Male out-migration

For households that experienced more challenges finding stable or suitable employment, many contributed to a growing trend of temporary or permanent male out-migration from drought-affected communities. Men frequently left villages for extended periods of time in search of employment opportunities, like construction jobs for infrastructure projects; some migrated to areas with more regular rainfall to settle on more productive land, sending money back to their families in their origin communities. In some cases, families relocated once men earned sufficient income in their new places of residence.

*When a man moves out of the community, he leaves his wife and children. Some men move out during times of hardships...During that time, they think of how they are going to take care of their children, so they leave in search of jobs...They send money* via *mobile transfer to take care of the children.* — Woman, FGD, Kalomo.

Some women perceived out-migrating men to have abandoned their families by permanently resettling in other communities, leaving their spouses to become the sole caretakers of the household and family. In these circumstances, children remained in their mother’s care, constraining employment prospects for women who suddenly absorbed more caregiving responsibilities. For these reasons, participants unanimously explained that female-headed households were among the most vulnerable to hardships in times of drought.

*I am the one who looks after the children. I do not have a husband around to help me with the children. I have to make sure my children go to school and eat.* — Woman, IDI, Mongu.

### Relationship conflict and dynamics

Marital relationships were challenged in times of drought, particularly when disagreements around household purchases could not be reconciled. Tightened household incomes required more joint decision-making in even small household purchases; routine purchases, like clothing and children’s toys, were seen as luxuries in the context of drought. As women became increasingly involved in household budgeting and financial decision-making, they reported more frequent quarrels with their partners about household purchases, particularly when disagreements arose regarding men’s discretionary use of income.

*If there’s no food in the home, then there will be disputes and quarrels between husband and wife because there is no lotion, soap, no clothes, nothing...Last year, I wanted to leave my marriage because of disputes. We never used to argue much, but now it has worsened due to drought.* — Woman, IDI, Kalomo.

Many women were overburdened by a perceived imbalance in household and income-generating responsibilities, feeling especially unsupported by sporadically employed or unemployed male partners. Participants explained that reduced employment prospects in agriculture were to blame for high rates of male unemployment, which many women suggested was the driver of truant behavior and alcohol use among men in their communities. Some women notably chided male partners who spent earned income on alcohol, leading to additional conflict in households.

*Upon harvesting your crops and making some sales, your husband will come up with an excuse that he wants to settle a debt for alcohol. You start fighting because there is no way your hard-earned money should be used to settle debt when it was supposed to help in the household.* — Woman, FGD, Mongu.

***“Looking at how things are now, it’s unlikely for me to go back to school”: Depletion of women’s social assets and heightened vulnerability.***

While drought affected all facets of life, the financial and social repercussions of drought, from increased breadwinning demands to heightened food insecurity, disproportionately impacted women. An immediate consequence of diminishing household incomes is the inability to afford school fees, which resulted in growing rates of school attrition during droughts. Even in circumstances where families could afford school fees or could rely on other relatives to offset these financial burdens, some caregivers withdrew their children from school, so they could support their families financially through labor.

*I wanted to rewrite the subjects that I failed in Grade 12, but that did not happen because there was no money. We did not harvest a lot of rice as planned because of drought.* — Woman, IDI, Mongu.

*I don’t think or see a way where I will go to school because my parents depend on farming, but in the last two years, they don’t get fertilizer...From primary to secondary school, they took me to school through farming, but looking at how things are now, it’s unlikely for me to go back to school. I am still dreaming of becoming a nurse.* — Woman, IDI, Mazabuka.

Many participants explained that children, particularly girls, from the most vulnerable households were sent to other communities for domestic work. Some women acknowledged that the financial insecurity propagated by drought forced girls into early marriages. While participants indicated child marriage was prevalent prior to drought, they explained that heightened poverty from drought perpetuated girls being married off by their parents or guardians because they are no longer able to care for them or are getting a financial return from the dowry.

*People used to get married at the right age, like 25 years and above. But this time, children as young as 12 years-old are forced into early marriages in order for their parents to survive.* — Man, FGD, Mazabuka.

Drought-induced financial insecurity prompted women, in some cases, to exchange or sell sex to support themselves and their families. Some participants explained how women would trade sex for food, money, or farming inputs. Female-headed households were perceived as particularly vulnerable, as many lacked the appropriate farming tools and inputs to support their own farms, which are traditionally owned and maintained by men.

*If she goes to an area to ask for food, when she returns, the man now starts asking her, “What if we agree like this?” [trade sex for food]. Because she wants to feed her children back home, she agrees. The people she asks also get tired and say, “You should be used” [have sex in exchange for food].* — Man, FGD, Kalomo.

***“You end up having another child, but to feed that child is a hustle”: SRH service provision and fertility trajectories in the context of drought.***

Participants described numerous barriers to accessing health services, specifically long distances to health facilities, lack of transportation, and commodity stockouts – all of which preceded, but were exacerbated by, droughts. Drought increased demand for health services as more suffered from malnutrition and disease, but accessibility to and quality of these services simultaneously decreased as households struggled to find income to pay for basic expenses, like food and school fees. Some women, for example, delayed antenatal care-seeking due to high service costs. Additionally, community members and health providers alike reported an increase in childbirths outside health facilities as health services became increasingly unaffordable and inaccessible during drought.

*I do not have an oxcart or other means of transportation, so, I really find it hard when my wife is due...We spend a lot of time trying to organize transport to go to the health facility, which is 2 h away. Meanwhile, my wife is in a lot of pain. Sometimes it takes about 3 h to get to the clinic, plus the running around. Our women go through a lot of complications because of this.* — Man, FGD, Mongu.

Family planning services were particularly susceptible to drought-related disruptions. Prior to drought, frequent contraception shortages at health facilities drove women to purchase family planning commodities from private sources like pharmacies. For women using shorter-acting hormonal methods, like injectables or pills, routine contraceptive purchases from private sources became increasingly unaffordable as household incomes shrank during prolonged periods of drought. Routine commodity stockouts at health facilities additionally disincentivized women from traveling long distances for follow-up contraceptive services, only to be turned away once at the clinic. This resulted in higher family planning service interruptions and contraceptive discontinuation rates.

*Sometimes we fail to go to the clinic to get injections or pills because of long distances and hunger. Due to these reasons, you may go to get pills on different days not described by the nurse, and in the end, you fall pregnant...You end up having another child, but to feed that child is a hustle.* — Woman, FGD, Senanga.

*There is shortage of injections, so if you do not have a K30 or K25, you will leave without getting the injection. You go there and find that it is finished, and it is only in a chemist where it is sold...They get pregnant because they do not have money, and they know that at the clinic it is free.* — Woman, IDI, Mazabuka.

*My husband will complain about me denying him of sex, but its really because I am scared to get pregnant, and when my medicine expires, they don’t usually have family planning at the clinic for 2 to 3 months* — Woman, FGD, Kalomo.

Drought, nonetheless, heightened demand for family planning services, as women and men’s fertility intentions shifted alongside dwindling household incomes. Participants overwhelmingly expressed preferences for smaller families, who could have their essential needs met, during times of drought. For many women, this motivated use and adherence to contraceptive methods, in spite of the barriers to SRH service provision, intensified during droughts.

*At this facility, we used to deliver for a lot of mothers, but the numbers have gone in terms of deliveries because people have opted not to have babies* — Health Provider, IDI, Kalomo.

*I have 4 children. I would like to have 6 children. Drought has made me change my mind about having 6 children. The 6 children I desire to have may not have enough food to eat. I may just leave them suffering.* — Woman, FGD, Mongu.

Despite dominant norms around parity and family sizes, men were largely supportive of women’s decisions to delay or conclude childbearing altogether. In few cases, however, gendered social obligations to produce children, coupled with childbearing demands of farming-based incomes, competed with the financial realities of drought, creating tension between men’s fertility aspirations and actual caregiving capabilities.

*I have 5 children...My desire was to have 5 more children...because I don’t know what is going to happen in future. Some of them might die. My desire was to have 10 children, so that some of them can help me because no one knows what the future holds.* — Man, FGD, Mongu.

*Having many children is for your own advantage because of the many jobs we have in this community, many children will be necessary. That is why we say,* ***“****Munwe Omwe Tupwai Njina” [translates to “One finger cannot crush lice”]. One person cannot solve all the problems.* — Man, FGD, Mazabuka.

***“Those who didn’t do conservation farming had nothing”: Solutions and challenges to climate adaptation.***

While participants described protracted financial challenges and poor health as a consequence of droughts, many also identified strategies they had adopted to adapt to an increasingly unpredictable, harsh climate. More climate-responsive farming practices were practiced, which resulted in more productive crop yields even in drier climates characterized by shorter, more infrequent rains. These practices include planting of early-maturing crops (i.e., beans, sunflowers), tilling land quickly, planting immediately upon first rains, and diversifying crop seeds. Nevertheless, stakeholders noted major gaps in uptake of new agricultural techniques, citing insufficient community engagement and limited climate forecasting capabilities as rate-limiting factors of adoption.

*People are still used to the traditional way of cultivation. When we had the drought, those who had done conservation farming had harvested a little. But those who didn’t do conservation farming had nothing. Completely nothing.* — NGO Manager, IDI, Choma.

NGOs (i.e., faith-based organizations, churches) and the public sector were described as having an active presence in the local climate response. Existing activities primarily focused on responding to the immediate needs of communities most-impacted by droughts and other extreme weather events (i.e., flash floods), including provision of food relief and other farming inputs (i.e., seeds, livestock) for income generation. Some capacity-building activities, including climate-responsive farming practices, and climate change education were available but on a smaller scale and only in specific communities. Key challenges to introducing and scaling up climate resilience interventions identified by stakeholders were limited inputs and non-earmarked budgets for climate resilience programming.

*There was an organization that came to sensitize us on a project about gardening...They gave us weed killer after being taught [how to use it], but they didn’t provide us with fertilizer...After you cultivate the fields in preparation for the rainy season, you’re supposed to plant with fertilizer. But the fertilizer only comes in February.* — Man, FGD, Kalomo.

*You have this hazard which has come, meaning you’re going to shift all the monies that you had put aside for developmental activities. Even for these sectors, for example, health and agriculture, they have their own plans. When you have floods, they take away the money from developmental programs. When you’re dealing with disasters, we are taught that you cannot budget for disasters. Of course, you cannot budget for them because you do not know the magnitude.* — DMMU Staff, IDI, Choma.

## Discussion

In this qualitative study, severe and persistent droughts aggravated underlying socio-economic and structural vulnerabilities for women and girls. Across communities, drought highlighted gender-based inequities in household asset management: while men and women equally described pressures to identify alternative sources of income when small-scale agriculture became untenable, women reported imbalanced expectations to generate income while fulfilling unchanging caregiving responsibilities at home. This was particularly pronounced in households where men struggled to find new employment prospects or out-migrated (either temporarily or permanently) for work, leaving their non-migrating household members (i.e., women and children) with limited financial or material support. A qualitative study examining the impact of flooding events on rural livelihoods in Zimbabwe similarly documented women’s heightened caregiving obligations as men pursued formal employment to recuperate lost income [38]. Importantly, the aforementioned study did not report increased demands on women to find employment, as observed in the present study. Evidence from Nigeria has illustrated how climate variability, specifically inconsistent rainfall and droughts, catalyzes male out-migration from rural agricultural communities to urban areas; in the process, women absorb a greater share of domestic responsibilities and workloads but with fewer resources than men, like farming inputs and deeds to land [39].

Direct economic consequences of drought, from diminishing household assets (i.e., livestock, income) to food insecurity, synergistically increased women’s risk to poor sexual and reproductive health outcomes. As household incomes dwindled, and women scrambled to provide sustenance for their families, their vulnerability transactional sex relationships (i.e., trading sex for food or income) or to early marriages was intensified. Numerous studies have linked food insecurity to heightened sexual risk among girls and women in sub-Saharan Africa [40, 41], though these associations are not explicitly interrogated in the context of drought or other extreme weather events. A nationally representative household survey in Lesotho reported significant associations of residence in drought-affected rural areas with early sexual debut and HIV prevalence, respectively, in adolescent girls [42]. Another pooled analysis of 19 population-based surveys in sub-Saharan Africa similarly showed that adolescent girls and unemployed women experienced higher rates of intimate partner sexual and physical violence in drought-affected communities [43]. A recent analysis of national vital statistics from 91 LMICs found that food insecurity mediated the association between drought and women’s HIV prevalence [44]. An advantage of the qualitative approach used in the current study is its capacity to situate intermediary variables, like early marriage or transactional sex, on the mechanistic pathway from food insecurity to sexual health outcomes in drought-affected areas.

A novel contribution of this qualitative study was its exploration of SRH needs and service provision in the context of drought. Findings reveal that SRH services became increasingly inaccessible during droughts, as dwindling incomes constrained resources and opportunities for seeking and obtaining healthcare. Women and facility-based providers explained how health services along the maternal health continuum, from antenatal care appointments to facility-based birthing services, were financially untenable during droughts: with minimal discretionary income for transportation and out-of-pocket expenses for facility-based services, women defaulted from antenatal care and opted for home births. Ethnographic, participatory studies in the Peruvian Amazon [45] and the Indian Sundarbans [46] have described how climate shocks, specifically heavy rainfall and flash floods, exacerbate underlying infrastructural barriers (e.g., roads, transport) to antenatal and post-partum service provision. Similar insights have been derived from health facility assessments and qualitative research in Bangladesh, where severe flooding rendered antenatal and reproductive health facilities inaccessible by transport [47, 48]. Drought-affected communities in Zambia, by comparison, reported financial, rather than ecological, disruptions to SRH service provision as a consequence of climate shocks: drought-induced financial insecurity limited women’s capacity to afford SRH services in the context of pre-existing unavailability (i.e., long distances to facilities from rural communities) and fledgling health systems infrastructure (i.e., supply stockouts).

Family planning and contraceptive services were uniquely impacted by drought in this study. Most interviewed women reported current contraceptive use, notably short-acting hormonal methods (i.e., injectables, pills), but many described barriers to use and continuation during drought. Stockouts at health facilities were frequently reported, diverting women away from public health facilities (where contraceptives were available for free) to private sources, like pharmacies, where contraceptives were available but sold at unsubsidized rates. Numerous studies have described challenges with contraceptive continuation and report high discontinuation rates in disaster and humanitarian contexts [49, 50], but few have described the impact of repeated climate shocks, like droughts, on contraceptive provision and use. Panel data from Tanzania showed that women experiencing income shocks from poor crop yields reported significantly higher rates of contraceptive uptake, albeit primarily traditional methods (e.g., rhythm method) [51]. Repeated household surveys in northern Ethiopia similarly showed steady increases contraceptive acceptability and fertility declines in drought- and famine-prone communities [52]. Another qualitative study from Ethiopia framed contraception as a mechanism for delaying childbearing during periods of drought [53]. None of these studies, importantly, has described how drought-induced economic shocks create barriers to contraception provision at the service-delivery point (facility-level) as well as continuation for the end-user (patient-level). Family planning provision remains a salient challenge in Zambia, where modern contraceptive prevalence among married women hovers around 48.5% – falling below the Family Planning 2020 commitment of 58% [54]. Drought, therefore, poses an exigent threat to SRH service-delivery in Zambia, where limited contraceptive method mix at health facilities, particularly in rural areas most impacted by drought, creates substantial barriers to women’s family planning use [55, 56].

Findings from this study join a burgeoning, albeit limited, body of evidence linking climate shocks to reduced fertility intentions in LMICs. Demographic studies in Indonesia [57] and across sub-Saharan Africa [51, 58, 59] consistently report exposure to extreme weather events and climate shocks is associated with reduced fertility desires, measured through self-reported changes ideal family size or waning desires for additional children. An ethnographic study in Ethiopia similarly illustrated how fertility intentions diminished in the context of persistent drought and famine, with participants citing concerns around child health and wellbeing in the face of harsh climactic and financial volatility [53]. Of note, interviewed men in this study, while expressing support for smaller families in times of drought, expressed future fertility intentions under more favorable economic and environmental circumstances. A qualitative study in rural Kenya displayed similar tensions between gendered fertility/virility expectations among men and the demands of large families on shrinking household resources in the context of climate change [60]. Declining fertility intensions among men and women competed with diminished access to health resources, including contraception provision, that could empower families to delay childbearing.

### Limitations

There are several limitations to this study. The condensed study implementation timeline could not accommodate purposive sampling of deviant or disconfirming cases, yielding a diverse study population with largely homogenous perspectives and experiences. Given the breadth of topics addressed in the field guides, sampling was insufficient for identifying participants with personal experiences across all thematic domains, limiting the depth with which specific topics (i.e., early marriage, transactional sex) could be fully interrogated. Additionally, only the consequences (rather than causes) of health systems factors that predated, but were aggravated in the context of, drought (i.e., commodity stockouts, transportation demands) could be investigated in this study. Credibility and confirmability of these findings, nonetheless, were enhanced through narrative consistency among participants and triangulation of interview findings across participant subgroups [61]. Qualitative data were only collected through IDIs and FGDs; inclusion of additional data collection methods, like direct observation of household farming activities or health facilities, may have generated additional insights supplementing the textual data presented in this study. Lastly, in light of the accelerated timeline for data collection, participant recruitment was restricted to five districts where rainfall anomalies were particularly pronounced. As such, narratives conveyed in IDIs and FGDs may have limited transferability to community members and stakeholders in districts impacted by extreme weather events other than droughts (e.g., flash flooding). Future studies should compare how variations climactic disturbances (i.e., flooding versus drought) differentially impact SRH service provision and access.

## Conclusions

**Fig. 3:**
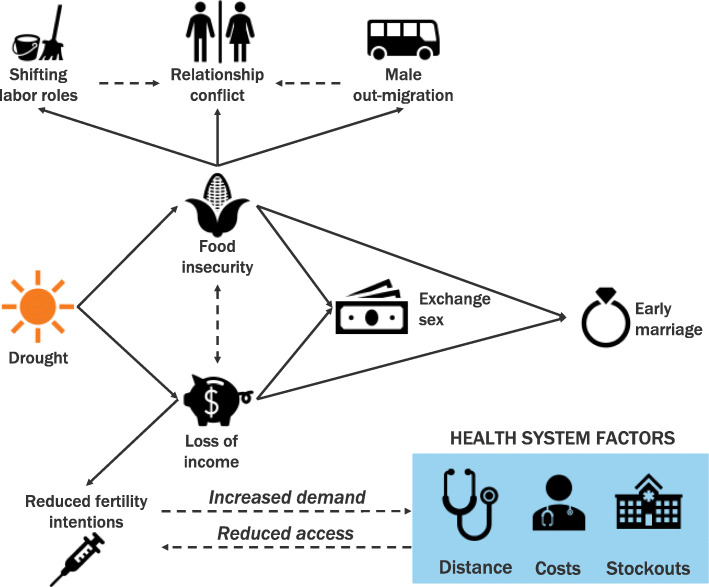
Pathways from drought to women’s vulnerability and sexual and reproductive health outcomes in Zambia, as mediated by food insecurity and loss of income

Drought-affected communities in Zambia are susceptible to overlapping economic instability and food insecurity, intensifying underlying social and structural vulnerabilities for women and girls to sexual health risks, from early marriage to transactional sex. While economic circumstances in times of drought increase demand for health services, in particular family planning, existing health resources compete with shrinking household incomes, rendering these services inaccessible to households most impacted by drought. These pathways are illustrated in Fig. 3. Existing interventions to support drought-affected communities offer some immediate relief but lack sustainable solutions for helping communities adapt to the consequences of climate change.

Policy solutions to foster climate resilience and mitigate challenges precipitated by persistent drought are presented in Table 2. To rectify the immediate SRH needs of women, GRZ should prioritize procurement and promotion of long-acting reversible contraceptive methods, which will mitigate care-seeking challenges reported by women while reducing commodity consumption strain on the health system. Task-shifting and increasing health workforce density, including investing in a community-based distributor cadre model for contraceptive provision, are other feasible, cost-effective solutions to increase provision of essential health services, particularly during extreme weather events [62]. In addition to immediate food and water relief, activities to catalyze agricultural output and community development during periods of drought include fertilizer and farming input subsidies; provision of start-up capital through low-interest loans for sustainable business development; and building efficacy and capacity for climate-response farming practices.

**Table 2:** Climate-related challenges and identified policy solutions from qualitative interviews with community members in drought-affected communities and key stakeholders

Domain	Challenges Identified	Policy Solutions
Agriculture	Poor crop yields due to rainfall anomalies	• Crop diversification, including hybrid seed introduction
		• Planting climate-resilient crops with early maturing seeds (e.g., legumes, sunflower)
		• Investments in alternative, renewable energy sources to reduce deforestation demands
		• Fertilizer and farming input subsidies
Economic development	Food insecurity, hunger, & malnutrition	• Food relief for the most vulnerable households
		• Start-up capital (via low-interest loans) for business ventures
		• Sink more boreholes for human and livestock consumption
SRH	Unaffordability & inaccessibility of SRH services	• Procurement and promotion of long-acting reversible contraceptive methods (i.e., Sayana Press)
		• Task-shifting to and expanded financing of non-facility-based health workforce cadres (i.e., community-based distributors)
		• Construction and staffing of additional rural health posts

## Data Availability

The data used and analysed during the current study are available from the corresponding author (MTM) on reasonable request.

## Abbreviations

DMMU: Disaster Management and Mitigation Unit
FGD: Focus group discussion
GRZ: Government of the Republic of Zambia
IDI: In-depth interview
LMIC: Low- and middle-income country
MoA: Ministry of Agriculture
MoH: Ministry of Health
NGO: Non-governmental organization
SRH: Sexual and reproductive health
WHO: World Health Organization
